# Nerolidol Attenuates Oxidative Stress, Inflammation, and Apoptosis by Modulating Nrf2/MAPK Signaling Pathways in Doxorubicin-Induced Acute Cardiotoxicity in Rats

**DOI:** 10.3390/antiox10060984

**Published:** 2021-06-21

**Authors:** Seenipandi Arunachalam, M. F. Nagoor Meeran, Sheikh Azimullah, Charu Sharma, Sameer N. Goyal, Shreesh Ojha

**Affiliations:** 1 Department of Pharmacology and Therapeutics, College of Medicine and Health Sciences, United Arab Emirates University, Al Ain 17666, United Arab Emirates; seenipandi@uaeu.ac.ae (S.A.); nagoormeeran1985@uaeu.ac.ae (M.F.N.M.); azim.sheikh@uaeu.ac.ae (S.A.); 2 Department of Internal Medicine, College of Medicine and Health Sciences, United Arab Emirates University, Al Ain 17666, United Arab Emirates; charusharma@uaeu.ac.ae; 3 Shri Vile Parle Kelavani Mandal’s Institute of Pharmacy, Dhule 424001, India; goyal.aiims@gmail.com

**Keywords:** acute cardiotoxicity, cardioprotective, doxorubicin, inflammation, nerolidol, oxidative stress, sesquiterpene

## Abstract

The clinical usage of doxorubicin (DOX), a potent anthracycline antineoplastic drug, is often limited by its cardiotoxic effects. Thus, for improving usage of DOX, the aim of this study was to assess the cardioprotective effects of nerolidol (NERO) in a rat model of DOX-induced acute cardiotoxicity and examine underlying molecular mechanisms that contribute to these effects. To induce acute cardiotoxicity male albino Wistar rats were injected with single dose intraperitoneal DOX (12.5 mg/kg). The rats were treated with NERO (50 mg/kg, orally) for five days. DOX-injected rats showed elevated levels of cardiac marker enzymes and enhanced oxidative stress markers along with altered Nrf2/Keap1/HO-1 signaling pathways. DOX administration also induced the activation of NF-κB/MAPK signaling and increased the levels and expression of pro-inflammatory cytokines (TNF-α, IL-6, and IL-1β) as well as expression of inflammatory mediators (iNOS and COX-2) in the heart. DOX also triggered DNA damage and apoptotic cell death in the myocardium. Additionally, histological studies revealed structural alterations of the myocardium. NERO treatment exhibited protection against the deleterious results of DOX on myocardium, as evidenced by the restoration of altered biochemical parameters, mitigated oxidative stress, inflammation, and apoptosis. The findings of the present study demonstrate that NERO provides cardioprotective effects against DOX-induced acute cardiotoxicity attributed to its potent antioxidant, anti-inflammatory, and antiapoptotic activities through modulating cellular signaling pathways.

## 1. Introduction

Chemotherapeutic agents have significantly improved the likelihood of survival among cancer patients [1]. Of the numerous chemotherapeutic agents, doxorubicin (DOX) also known as Adriamycin belongs to anthracycline class of cytotoxic antibiotic, which is extensively used in the treatment of multiple types of malignancies sarcoma, carcinoma, lymphoma and leukemia [2]. However, the clinical use of DOX is often limited due to its severe adverse effects, including neurological disturbances, bone marrow aplasia and cardiotoxicity [3]. The appearance of acute cardiotoxicity necessitated the need for effective agents to mitigate DOX-induced acute cardiotoxicity [4].

To reduce the occurrence of acute cardiotoxicity induced by DOX, numerous protective strategies have been developed. This includes the development of improved dosage forms of DOX itself, as well as the development of agents that mitigate oxidative stress, inflammation, and apoptosis. This leads to biochemical, molecular, structural and histological alterations and appear as common pathogenic events in DOX-induced acute cardiotoxicity [5,6]. Numerous antioxidants, anti-inflammatory and antiapoptotic agents of natural and synthetic origins, have been shown to exert protective effects in preclinical models of DOX-induced cardiotoxicity. Among numerous synthetic antioxidants, dexrazoxane, a derivative of the chelating agent ethylenediaminetetra-acetic acid appears one of the promising agents for the treatment of DOX-induced cardiotoxicity. Though, synthetic antioxidant, dexrazoxane has been found potentially beneficial in reducing the generation of oxygen radical species [7]. But, it’s like other synthetic antioxidants possesses adverse effects. Thus, many naturally occurring plant-derived secondary metabolites, termed phytochemicals, have received enormous attention for their potential chemopreventive, chemotherapeutic, and cardioprotective benefits [8,9,10].

In recent years, numerous phytochemicals have been showed to exert chemopreventive, chemotherapeutic, chemosensitizing, and cardioprotective properties that mediate between antioxidant and anti-inflammatory properties, along with cell death pathway modulation [8,9,10,11]. Accumulative studies over the years demonstrated that numerous mechanisms are involved in DOX-induced cardiotoxicity. One of the important mechanisms is the overproduction of free radicals, which leads to the exhaustion of endogenous antioxidant defense, lipid peroxidation, induction of inflammatory mediators, DNA damage, programmed cell death, and necrosis in an orchestrated manner [12,13]. DOX has been also shown to elicit a significant rise in the levels of pro-inflammatory cytokines and inflammatory mediators in the myocardium [14]. Nuclear factor kappa-B (NF-κB) activation and mitogen-activated protein (MAPK) signaling is known to trigger apoptosis by activating a pro-apoptotic event [15]. DOX also showed to induce apoptosis via c-Jun N-terminal kinases (JNKs) and MAP kinase signaling pathways [16].

Additionally, NF-κB inversely regulates the transcription and activities of nuclear factor erythroid 2-related factor 2 (Nrf2), a transcription factor. Nrf2, a master regulator of oxidative stress, is known to orchestrate redox defense mechanisms in the event of oxidative stress via the augmentation of endogenous antioxidant defense mechanisms and the activation of hemeoxygenase-1 (HO-1) [17,18]. Nrf2 and its principal negative regulator, the E3 ligase adaptor Kelch-like ECH-associated protein 1 (Keap1), potentially appears to be a therapeutic target to mitigate DOX-induced cardiotoxicity through sustaining antioxidant defense mechanisms at cellular levels in the myocardial cells. To curb the multifactorial nature of DOX-induced cardiotoxicity, the naturally occurring agents appears to mitigate oxidative stress, inflammation, and apoptosis, which could be important for mitigating pathogenic events of oxidative stress, inflammation and apoptosis [19]. Many antioxidant, anti-inflammatory and antiapoptotic agents have been shown to improve endogenous antioxidants, as well as reduce inflammatory and apoptotic cascades by promoting cell survival and cell signaling pathways [19].

In recent years, many naturally occurring phytochemicals have been revealed to mitigate DOX-induced cardiotoxicity [20,21,22]. Among numerous phytochemical classes, terpenes and terpenoids have garnered attention for their potential health benefits and therapeutic and preventive potential in cancers and cardiovascular diseases [9,10]. One of the sesquiterpenes, nerolidol (NERO), has specifically received interest for its potential benefits in cancers and cardiovascular diseases [23]. NERO is abundantly found in the essential oils of many ornamental, medicinal, and edible or dietary plants [23]. It is one of the common components of Oolong tea (a traditional semi-oxidized Chinese tea) and is known to contribute to the floral aroma to tea, the world’s most popular beverage [24]. It is also present in Madeira wines from Portugal [25]. It is characterized with a floral odor and contribute to flavor of fruits including kiwis and strawberries. It is used as a flavor enhancer in foods, cosmetics, and beverages [26,27].

NERO is approved by the Food and Drug Administration (FDA) and listed in catalogue of substances “generally recognized as safe” (GRAS) for use in food and beverages [23]. NERO also possesses antimicrobial, antibiofilm, antioxidant, antiparasitic, anti-nociceptive, anti-inflammatory, and anticancer properties [23,28]. It has been shown to activate antioxidant signaling pathways and attenuate inflammatory and apoptotic signaling pathways, as well as exert cytoprotective properties by improving antioxidant defense [29] and exerting anti-inflammatory effects [23,30,31]. Recently, NERO has been shown to attenuate oxidative stress, inflammation, fibrosis, and apoptosis in cardiotoxicity induced by cyclophosphamide, a chemotherapeutic agent [32], isoproterenol-induced myocardial infarction [33], and modulate contractile function of the atrium [34]. Additionally, NERO has been shown effective as an anticancer agent due to its potential to modulate important molecular targets of cell survival and proliferation [35], as well as act as a chemosensitizer in cancer cells [36,37,38]. NERO has also been shown to enhance the efficacy of DOX in breast cancer cells [36] and improve DOX efficacy in sensitive and partly resistant lymphoblast and ovarian cancer cells [39].

NERO showed to improve DOX accumulation in cancer cells without affecting DOX concentration in hepatocytes [37]. It also showed to exert an inhibitory effect on carcinogenesis of the large intestine [38]. The potent cardioprotective, chemopreventive, anticancer, and chemosensitizing property of NERO needs to be further evaluated for its potential in DOX-induced acute cardiotoxicity given the preclinical evidence that it synergistically interacts with DOX in cancer chemotherapy. The aim of this study was to evaluate the cardioprotective effect of NERO in acute cardiotoxicity. Our findings further substantiate the fact that DOX is used more than cyclophosphamide in a wide variety of cancers. Moreover, NERO was found not to affect the clinical efficacy of DOX, as it exerted synergistic effects on resistant cancer cells [36,37,38,39].

In view of this, it is important to evaluate the cardioprotective potential of NERO against DOX-induced acute cardiotoxicity in rats. More specifically, to better understand the role of NERO in DOX-induced acute cardiotoxicity, we investigated the effect of NERO on antioxidant defense, inflammatory markers, and apoptosis. As such, we elucidated the molecular mechanisms of cardioprotection attributed by the modulation of cell signaling pathways.

## 2. Materials and Methods

### 2.1. Drugs, Chemicals and Kits

The test compound, Nerolidol was purchased from Sigma Aldrich, St. Louis, MO, USA. Doxorubicin was purchased from EBEWE Pharma, Unterach, Mondseestrasse, Austria. The commercially available kits for the biochemical estimations were obtained from Sigma Aldrich, St. Louis, MO, USA and Abcam, MA, USA.

### 2.2. Experimental Animals

The animal research protocols and procedures were approved by the United Arab Emirates University Animal Ethics Committee (ERA_2019_5919). Male adult albino Wistar rats in the weight range of 180–190 g were obtained from the University Animal Research Facility at the College of Medicine and Health Sciences (CMHS), United Arab Emirates University, UAE. The animals were kept in polypropylene animal cages (47 × 34 × 20 cm), which contained a layer of husk that was replaced every 24 h. The animal facility maintained at standard animal housekeeping, including a photoperiod of 12 h light/dark cycle, a temperature of 22 °C, and a humidity of 45–55%. The animals were fed with a standard rodent chow diet procured from the National Feed and Flour Production and Marketing Company, LLC., Abu Dhabi, UAE. The animals had free access to water ad libitum.

### 2.3. Induction of Cardiotoxicity in Rats

To induce acute cardiotoxicity in rats, a single dose of DOX (12.5 mg/kg body weight) was intraperitoneally injected [6]. The changes in the levels of myocardial enzymes were diagnostic biomarkers of DOX-induced cardiotoxicity in rats.

### 2.4. Experimental Design

Rats were randomly allocated into four experimental groups, each containing eight rats. Group I: normal control rats; group II: rats orally treated with NERO (50 mg/kg body weight) and dissolved in olive oil daily for five days; group III: rats intraperitoneally injected with DOX (12.5 mg/kg body weight) to induce acute cardiotoxicity; group IV: rats administered with a single intraperitoneal dose of DOX (12.5 mg/kg body weight) and orally treated with NERO (50 mg/kg body weight) for five days. After the treatment period (i.e., on sixth day), all rats were anaesthetized with pentobarbital sodium (60 mg/kg body weight) and then killed via cervical decapitation. The blood samples were collected and centrifuged at room temperature for 10 min at 4000 rpm. The supernatant (serum) was collected and frozen at −80 °C until further analysis. Hearts were collected and snap frozen in liquid nitrogen before being stored at −80 °C for biochemical analysis. Samples were also placed in 10% neutral buffered formalin and stored at 4 °C for histological study.

### 2.5. Assay of Cardiac Marker Enzymes

The levels of serum creatine kinase (CK) and lactate dehydrogenase (LDH) was assayed using VetTest 8008 Chemistry Analyzer (UK). The levels of serum troponin-T were estimated using the commercial kit obtained from MyBiosource International, San Diego, CA, USA.

### 2.6. Estimation of Lipid Peroxidation and Antioxidants

The levels of malondialdehyde (MDA) in the heart homogenate were determined using a commercially available kit (North West Life science, WA, USA). The data obtained are expressed as μM/mg protein. The concentrations of superoxide dismutase (SOD) and catalase/glutathione contents were estimated using commercially available kits following the manufacturer’s instructions (Sigma Aldrich, St. Louis, MO, USA; Cayman Chemical Company, Ann Arbor, MI, USA).

### 2.7. Enzyme Linked Immunosorbent Assay (ELISA)

The levels of proinflammatory cytokines in the heart such as tumor necrosis factor-α (TNF-α), interleukin-6 (IL-6), and interleukin-1β (IL-1β) were measured in the myocardial homogenates using ELISA kits (MyBiosource International, San Diego, CA, USA).

### 2.8. Western Blot Analysis

Heart tissues were homogenized in a RIPA buffer with protease and phosphatase inhibitors (Sigma Aldrich, MO, USA) and the homogenates were centrifuged at 14,000 rpm (4 °C for 30 min). The supernatant was collected and mixed well with a 4× laemmlli buffer (Bio Rad, Hercules, CA, USA) and 2-mercaptoethanol (Sigma Aldrich, St. Louis, MO, USA). By using the gel electrophoresis, equal amounts of protein were separated and transferred onto PVDF membranes (Thermo Fisher Scientific, Waltham, IL, USA). The PVDF membrane was incubated at 4 °C overnight. It contained primary antibodies against inducible nitric oxide synthase (iNOS) (anti-rabbit; Sigma Aldrich, St. Louis, MO, USA), cyclooxygenase-2 (COX-2), Bcl2 associated X protein (Bax), B-cell lymphoma-2 (bcl2), B-cell lymphoma extra-large (Bcl-xL), active caspase-3, Nrf2, cytochrome-C, keap-1, HO-1, IκB-α, *p*-IκB-α, t-P^38^, *p*-P^38^, *p*-IKKα, apoptotic protease activating factor 1 (APAF-1) (anti-rabbit and mouse, Abcam, Cambridge, MA, USA), t-IKKα, t-Jun-amino-terminal kinase (t-JNK), p-Jun-amino-terminal kinase (*p*-JNK), p-67 phox, cleaved poly (ADP-ribose) polymerase (cleaved PARP) (Santa Cruz Biotechnology, Dallas, TX, USA), superoxide dismutase 1 (SOD1), superoxide dismutase 2 (SOD2), active caspase-9 (Cell Signaling Technology, Danvers, MA, USA), nuclear factor kappa-B-p65 (NF-κB-p65), phospho nuclear factor kappa-B-p65 (*p*-NF-κB-p65), and β-actin (anti-mouse; Millipore, Burlington, MA, USA). The PVDF membrane was again incubated for 1 h with the corresponding secondary antibodies. The protein expressions were visualized using a chemiluminescence West Pico kit (Thermo Fisher Scientific, Waltham, MA, USA). To measure the intensity of the signals, we conducted a densitometric analysis using Image J software (National Institutes of Health, Bethesda, MD, USA).

### 2.9. Histopathological Evaluation

Heart tissues were fixed in formalin for a week. Then, the formalin fixed tissues were processed following a series of exchanges in different solvents. Finally, they were embedded in the paraffin wax. The formalin-fixed paraffin embedded tissues were serially sectioned in 5–10 μm thickness using a microtome (Leica Biosystems, Nussloch, Germany). The tissue sections were stained with hematoxylin and eosin (H&E). Thereafter, these were mounted on gelatin coated glass slides. The mounted sections were visualized under a light microscope BX41 obtained from Olympus, Center Valley, PA, USA and images were captured for histopathological assessment.

### 2.10. Estimation of Protein Concentration

The total amount of protein in the samples were determined using the Pierce^TM^ BCA protein assay kit (Thermo Fisher Scientific, Waltham, IL, USA) and according to the manufacturer’s instructions.

### 2.11. Statistical Analysis

The results are shown as mean ± standard error of the mean (SEM). The differences among groups were statistically analyzed via one-way analysis of variance (ANOVA) followed by a Duncan’s multiple range test (DMRT). The data were analyzed using IBM SPSS statistics v24.0 (SPSS Inc., Chicago, IL, USA). The criterion of statistical significance was set at the *p* value less than 0.05.

## 3. Results

### 3.1. Nerolidol (NERO) Reinstates Myocardial Marker Enzymes and Doxorubicin (DOX)-Induced Changes in the Myocardial Architecture in Rats

**Figure 1:**
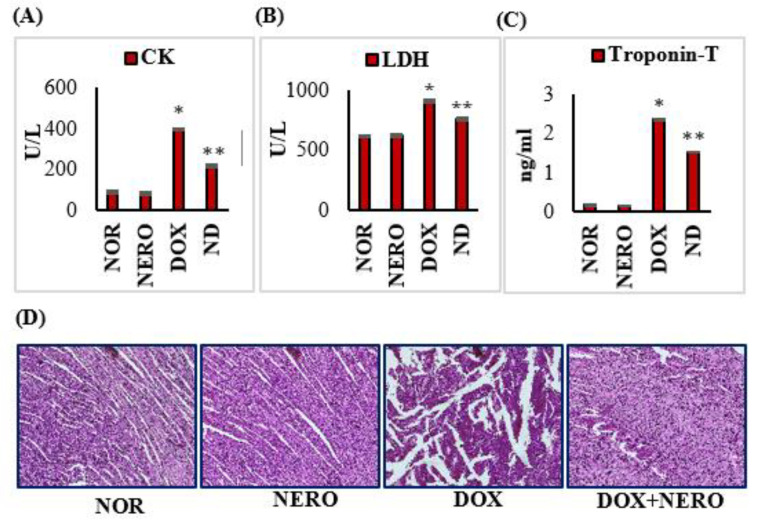
(**A**–**C**) Effect of NERO on myocardial injury markers; columns do not share a common symbol (*, **) and differ significantly from each other (* *p* < 0.05 vs. normal control, ** *p* < 0.05 vs. DOX control). (**D**). Histopathology of the myocardium: Normal control rat’s heart has normal architecture of the myocardium; NERO alone treated rat’s heart also shows normal intact muscle fibers without any pathological changes; DOX-injected rat’s heart shows extensive muscle fiber degradation with inflammatory cells; NERO treated DOX-challenged rats shows a reduced muscle fiber degradation without inflammatory cells. CK- creatine kinase; LDH- lactate dehydrogenase; NOR-normal; NERO-nerolidol; DOX- doxorubicin; ND- rats administered with a single intraperitoneal dose of DOX and orally treated with NERO.

DOX-injected rats showed a significant (*p* < 0.05) increase in the serum levels of creatine kinase (CK), lactate dehydrogenase (LDH), and troponin-T compared to the normal control rats. However, treatment with NERO prevented a cardiomyocytes injury-induced release of CK, LDH, and troponin-T into the serum in DOX administered rats compared to DOX control rats. The hearts in normal and NERO treated rats showed normal myocardial histoarchitecture with no changes in the cardiomyocytes. This finding suggests that there are no adverse effects of NERO at this particular dose and that it is devoid of any adverse effects on cardiomyocytes. Rats injected with DOX showed an intense degradation of muscle fibers with clear signs of inflammation. On the other hand, DOX-injected rats treated with NERO (ND) were revealed to have reduced muscle fiber degradation with negligeable inflammatory cells (Figure 1).

### 3.2. NERO Attenuates DOX-Induced Oxidative Damage via Activation of Nuclear Factor Erythroid 2-Related Factor 2 (Nrf2) Signaling Pathway

**Figure 2:**
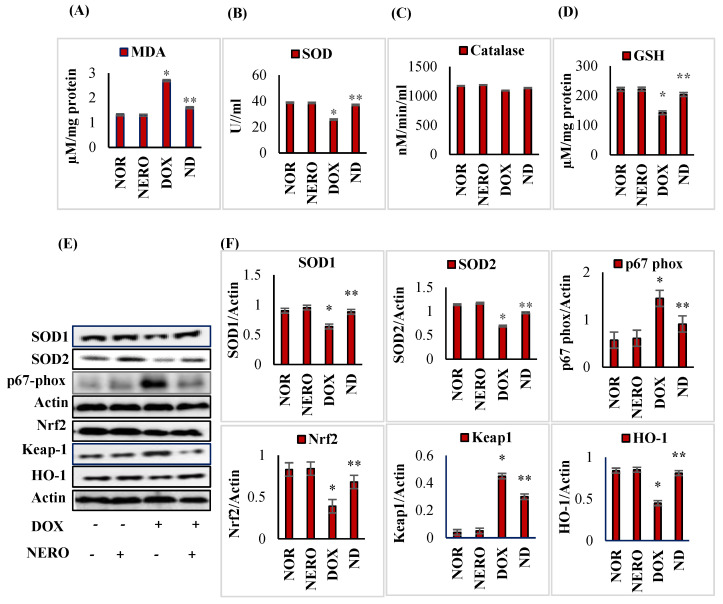
(**A**–**D**) Effect of NERO on lipid peroxidation and oxidative stress. Lipid peroxidation and antioxidant status in the heart. Each column is mean ± SEM for eight rats in each group; columns do not share a common symbol (*, **) and differ significantly with each other (* *p* < 0.05 vs. normal control, ** *p* < 0.05 vs. DOX control). (**E**,**F**). Western immunoblot analysis and densitometric analysis for SOD1, SOD2, p67-phox, Nrf2, HO-1, and Keap-1. Values are expressed as mean ± SEM (*n* = 3); columns do not share a common symbol (*, **) and differ significantly with each other (* *p* < 0.05 vs. normal control, ** *p* < 0.05 vs. DOX control). MDA- malondialdehyde; SOD 1- superoxide dismutase 1; SOD 2- superoxide dismutase 2; HO-1-heme oxygenase-1; GSH-reduced glutathione; Nrf 2- Nuclear factor erythroid 2-related factor 2; NOR-normal; NERO-nerolidol; DOX- doxorubicin; ND- rats administered with a single intraperitoneal dose of DOX and orally treated with NERO.

DOX administered rats showed a significant (*p* < 0.05) increase in malondialdehyde (MDA) content with a significant (*p* < 0.05) decrease in the activities/concentrations of superoxide dismutase (SOD), catalase, and reduced glutathione (GSH), at least when compared to the normal control rats. Moreover, DOX-injected rats showed a significant (*p* < 0.05) decrease in the expressions of myocardial proteins, heme oxygenase-1 (HO-1) and Nrf2 demonstrated a significant (*p* < 0.05) increase in the expression of Kelch-like ECH-associated protein 1 (Keap-1) in the myocardium compared to the normal control rats. However, rats treated with NERO showed a significant (*p* < 0.05) reduction in MDA levels and a significant (*p <* 0.05) increase in the activities/concentration of SOD, catalase, and GSH, at least when compared to the DOX control rats. NERO treatment also showed a significant (*p <* 0.05) increase in the expressions of myocardial proteins. HO-1 and Nrf2 had a significant (*p <* 0.05) decrease in the myocardial protein expressions of Keap-1 in DOX-injected rats compared to DOX control rats (Figure 2).

### 3.3. NERO Treatment Attenuates Induction and Release of Pro-Inflammatory Cytokines

**Figure 3:**
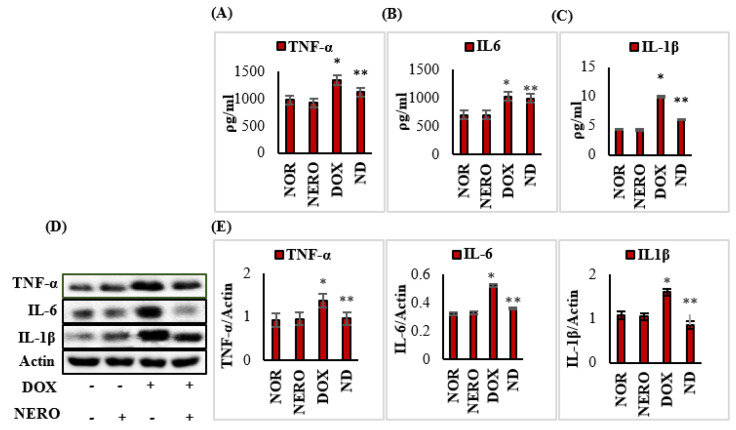
(**A**–**E**) Levels/expressions of TNF-α, IL-6, and IL-1β in the myocardium. Each column is mean ± SEM for eight rats in each group for ELISA and 3 rats in each group for immunoblotting. Columns do not share a common symbol (*, **) and differ significantly with each other (* *p* < 0.05 vs. normal control, ** *p* < 0.05 vs. DOX control). Representative images of Western immunoblot analysis and densitometric analysis for TNF-α, IL-6, and IL-1β in the myocardium. TNF-α- tumor necrosis factor α; IL-6- interleukin 6; IL-1β- interleukin 1β; NOR-normal; NERO-nerolidol; DOX- doxorubicin; ND- rats administered with a single intraperitoneal dose of DOX and orally treated with NERO.

The levels and expressions of tumor necrosis factor-α (TNF-α), interleukin-6 (IL-6), and interleukin-1β (IL-1β) were significantly (*p* < 0.05) increased in DOX-injected rats compared to normal control rats. However, NERO treatment exhibited a significant (*p* < 0.05) reduction in the levels and expressions of TNF-α, IL-6, and IL-1β in DOX-injected rats when compared to rats administered with just DOX (Figure 3).

### 3.4. NERO Attenuates Inflammatory Mediators and Modulates Altered Nuclear Factor Kappa-B (NF-κB)/Mitogen-Activated Protein Kinase (MAPK) Signaling Pathways

**Figure 4:**
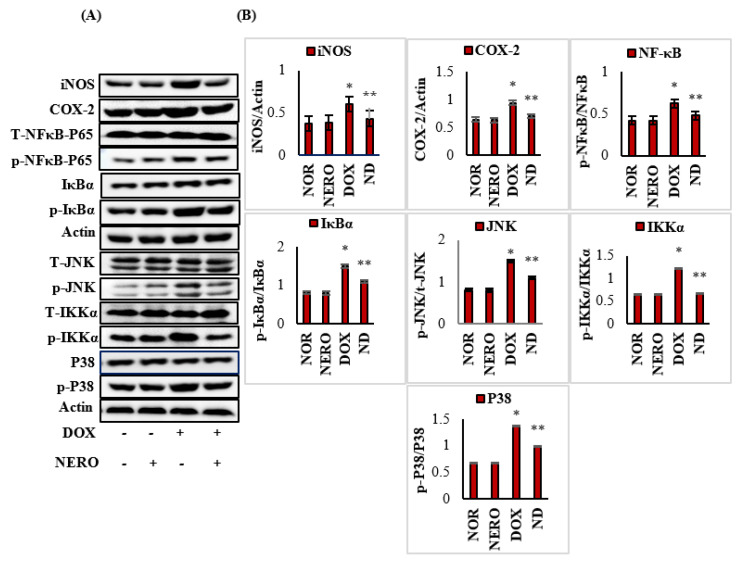
(**A**,**B**) Western immunoblotting and densitometric analysis for iNOS, COX-2, NF-κB-P65, *p*-NF-κBP65, IκBα and *p*-IκBα, t-IKKα, *p*-IKKα, t-JNK, *p*-JNK, P38, and *p*-P38. The values are expressed as the mean ± SEM (*n* = 3); columns do not share a common symbol (*, **) and differ significantly with each other (* *p* < 0.05 vs. normal control, ** *p* < 0.05 vs. DOX control).iNOS-inducible nitric oxide synthase; COX-2- cyclooxygenase-2; *p*-NF-κB-p65- phospho nuclear factor kappa-B-p65; t-JNK- t-Jun-amino-terminal kinase; *p*-JNK- p-Jun-amino-terminal kinase; *p*-IKKα- IκB kinase-*α*; P38-tumor protein; p-P38-phosphorilated p38; NOR-normal; NERO-nerolidol; DOX- doxorubicin; ND- rats administered with a single intraperitoneal dose of DOX and orally treated with NERO.

DOX-injected rats showed a significant (*p* < 0.05) increase in the expression of inflammatory mediators, inducible nitric oxide synthase (iNOS) and cyclooxygenase-2 (COX-2), NF-κB; *p*-NFκB-p65, IκB kinase-*α (p*-IKKα), and *p*-IκBα), and MAPK (*p*-P^38^ and c-Jun N-terminal kinases (*p*-JNK) in myocardium when compared to the normal control rats. NERO treatment, however, significantly (*p* < 0.05) decreased the expression of these proteins in DOX-injected rats, at least when compared to control rats that just had DOX administered (Figure 4A,B).

### 3.5. NERO Prevents DOX-Triggered DNA Damage and Apoptosis

**Figure 5:**
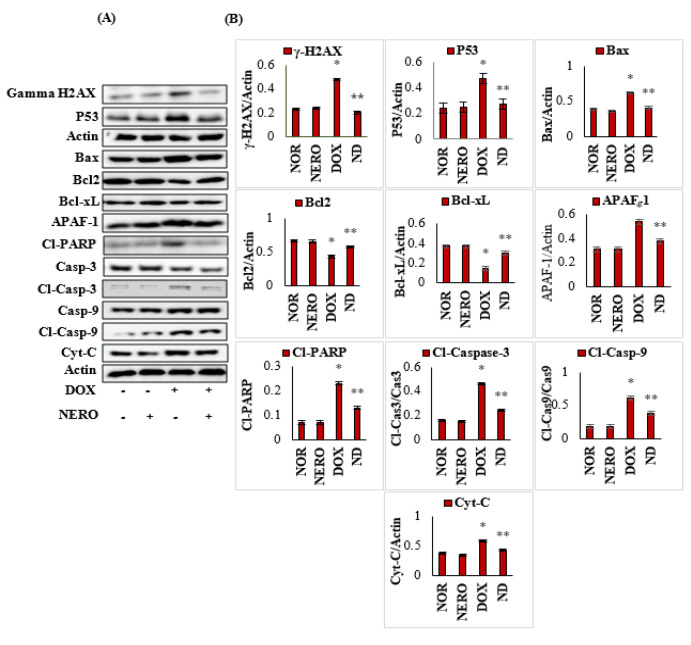
(**A**,**B**). Representative images of Western immunoblot analysis and densitometry for γ-H2AX, P53, Bax, Bcl2, Bcl-xL, active caspase-3, active caspase-9, APAF-1, cleaved PARP, and cytochrome-C. The values are expressed as the mean ± SEM (*n* = 3); columns do not share a common symbol (*, **) and differ significantly with each other (* *p* < 0.05 vs. normal control, ** *p <* 0.05 vs. DOX control). H2AX- H2A histone family member X; Bcl-2- B-cell lymphoma 2; Bax- Bcl2-associated-X- protein; Bcl-xL- B-cell lymphoma extra-large; P53- tumor protein; APAF-1- apoptotic protease activating factor 1; Cyt-C- cytochrome-C; CL-PARP-cleaved poly (ADP-ribose) polymerase; Casp-3-caspase 3; Cl-Casp-3- cleaved caspase-3; Casp-9-caspase 9; Cl-Casp-9- cleaved caspase-9; NOR-normal; NERO-nerolidol; DOX- doxorubicin; ND- rats administered with a single intraperitoneal dose of DOX and orally treated with NERO.

DOX-injected rats showed a significant (*p* < 0.05) increase in the expression of proapoptotic myocardial proteins, such as H2A histone family member X (γ-H2AX), Bcl2-associated-X- protein (Bax), tumor protein (P53), apoptotic protease activating factor 1 (APAF-1), cleaved poly (ADP-ribose) polymerase (cleaved PARP), cleaved caspase-9, and cleaved caspase-3 and cytochrome-C (Cyt-C). This was connected to a significant (*p* < 0.05) reduction in the expressions of B-cell lymphoma 2 (Bcl-2) and B-cell lymphoma-extra large (Bcl-xL) when compared to normal control rats. NERO treated rats exhibited a remarkable decrease in proapoptic proteins and induction in the expression of antiapoptotic proteins. This finding revealed its effect on cell death signaling markers in rats administered with DOX (Figure 5A,B).

## 4. Discussion

The results in the present study demonstrate the cardioprotective properties of NERO against DOX-induced acute cardiotoxicity. This was evidenced by the salvage of histopathology, restoration of myocyte injury markers and inhibition of lipid peroxidation. Further, this was substantiated by inhibition of oxidative stress, inflammation and apoptosis, mediating suppression of the Nrf2/Keap1 and NF-ĸB/MAPK signaling mechanisms.

Acute or chronic cardiotoxicity may appear after chemotherapy with DOX. The standard diagnostic biomarkers of myocardial cell injury in acute cardiotoxicity are troponins (T and I), CK, and LDH [40,41]. A remarkable rise in these cardiac diagnostic markers in rats injected with DOX demonstrate the deleterious effects of DOX on cardiomyocytes. Interestingly, treatment with NERO appeared to protect the myocardium, as evidenced by a reduced leakage of cardiac troponins, CK, and LDH into the circulation. The reduced leakage of myocyte injury markers concomitant to histopathological salvage of cardiomyocytes are ascribed to the potent membrane that stabilize NERO property. This fact further supports the reduction of the lipid peroxidation initiated by DOX-induced free radicals.

DOX-induced free radicals and consequent lipid peroxidation are important pathogenic events in myocardial cellular injury. The excessive generation of free radicals due to the increased oxidative metabolism and limited availability of antioxidant defense makes the myocardium more susceptible to DOX-induced cardiotoxicity [42]. The depletion of antioxidants, SOD, catalase, and GSH concomitant to lipid peroxidation after DOX injections demonstrates the occurrence of oxidative damage. Moreover, this reflects the generation of reactive oxygen species (ROS), which surpasses the antioxidant defense mechanisms [43]. Increased utilization of antioxidants for scavenging ROS might be the reason for declined antioxidant status [44]. Treatment with NERO in DOX-induced rats resulted in the normalization of SOD, catalase, and glutathione, as well as the inhibition of lipid peroxidation reflected by the reduced formation of MDA. Here, we demonstrated the antioxidant property of NERO and our findings were in agreement with previous studies wherein NERO found a well-known free radical scavenger and prevented lipid peroxidation and oxidative stress in the heart [32], liver [45] brain [31], and lungs [46].

The myocardial cells are equipped with Nrf2, a widely distributed antioxidant defense system that negatively regulates oxidative stress in tissues and curbs the occurrence of oxidative stress. In basal conditions, Nrf2, a redox-active signal protein, coexists with Keap1, a scaffold protein, for the ubiquitination and degradation of Nrf2. In cellular responses to oxidative stress, it undergoes dissociation and immediately translocates into the nucleus. This leads to the augmentation of antioxidant defense, which mediates the upregulation of detoxifying enzymes and phase II enzymes including SOD, glutathione cycle enzymes, NAD (*p*) H: quinone oxidoreductase (NQO-1), UDP-glucuronosyl transferases (UGTs), and heme oxygenase-1 (HO-1) [47]. DOX-induced cardiotoxicity has shown the suppression or inactivation of Nrf-2, and there is a resultant reduction in endogenous antioxidant enzyme levels and myocardial expression of HO-1. In the present study, NERO treatment significantly enhanced the activation of Nrf2/Keap1 signaling, which upregulated the endogenous antioxidant response in the myocardium. Our findings aligned with those from previous studies, wherein NERO was shown to attenuate cyclophosphamide-induced neuroinflammation, cognitive impairment [48], hepatic inflammation [45], colon inflammation [49], and acute lung-injury [50], all of which mediated Nrf2 upregulation. Regarding the interaction of NERO with the Nrf2 protein, in silico studies have shown that NERO with hydrogen bonds and hydrophobic interactions can bind the active binding site of the Nrf2 protein in its catalytic domain [48].

Myocardial inflammation is a crucial pathological alteration following DOX-chemotherapy [6]. DOX elicits inflammatory responses in myocardium by activating NF-κB signaling, contributing to the secretion of pro-inflammatory cytokines (IL-6, TNF-α, and IL-1β), and regulates inflammatory mediators (COX-2 and iNOS), which participates in inflammation [51,52]. NF-κB is a key player in the institution and propagation of inflammatory processes in an orchestrated manner, especially following the upregulation of inflammatory mediators and proinflammatory cytokines. The degradation of IκBα results in the nuclear translocation of NF-κB, wherein it triggers the synthesis of pro-inflammatory cytokines [43]. Treatment with NERO suppressed the induction of inflammatory mediators, COX-2, and iNOS, as well as the synthesis of proinflammatory cytokines and NF-κB, the latter of which attributes to its anti-inflammatory actions in myocardial inflammation. Additionally, in earlier studies, NERO attenuated myocardial inflammation and was induced by other chemotherapeutic drugs.

In addition to NF-ĸB, the MAPK family is a key regulator of signal transduction in cellular proliferation, differentiation, and apoptosis along with their primary role in regulating inflammatory mediators [53]. The JNK and p38 kinases upon inflammatory stimuli and mediator activation play critical roles in eliciting oxidative stress and inflammation in DOX-induced cardiotoxicity [16]. Considering the role of NF-ĸB/MAPK signaling suppression in DOX-induced cardiotoxicity, the present study investigated the ability of NERO against DOX-induced inflammatory events by assessing the components of NF-ĸB/MAPK signaling. In the present study, NERO treatment was observed to attenuate altered NF-ĸB/MAPK signaling in DOX-induced myocardial inflammation. This was concomitant to the inhibition of inflammatory mediators and histological salvages of the cardiomyocytes in DOX-challenged rats. In addition to the antioxidant properties, NERO potentially interacts with the catalytic ligand binding domain of NF-κB p65 by forming hydrophobic interactions to amino acid residues. This is believed to attribute to another mechanism that has an anti-inflammatory role [32]. The inhibitory action of NERO on NF-κB interferes with the positive feedback loop and modifies the target genes. This is the main reason for its cardioprotective effect [32].

Apart from oxidative stress and orchestrated inflammation events, apoptotic cell death of cardiomyocytes induced by DOX has been well reported in DOX-induced cardiotoxicity [54,55,56,57]. The onset and propagation of oxidative stress induced by DOX is known to elicit cardiac mitochondrial dysfunction and damage the cardiomyocytes [58]. DOX directly targets mitochondria through intrinsic apoptotic pathways and mitochondrial membrane alterations via cardiolipin binding involved in the functional changes of various mitochondrial parameters and the activities of respiratory chain enzymes [59]. Administering DOX induces protein ataxia-telangiectasia mutated (ATM) activation followed by DNA double strand breaks (DSBs), as well as the phosphorylation of p53 and H2AX [54,55]. Moreover, ATM is an apoptotic modulator during DNA damage responses (DDR) via p53 activation [60]. The myocardial protein expression of γ-H2AX is a marker of DSBs and a primary signal for chromatin responses to cardiac DNA damage in DOX-administered rats [56,57]. The downregulation of phosphorylation of p53 and H2AX following NERO treatment indicates its potential effect on apoptotic cell death elicited by DOX in cardiomyocytes.

The onset of apoptosis is regulated by apoptotic machinery, which involves caspases (caspase-3 and 9) and members of the Bcl-2 family, which are crucial meditators of the intrinsic pathway of cell death involving pro-apoptotic signals of Bax and anti-apoptotic signals of Bcl-2 [61]. DOX-induced ROS production induces cell death by inhibiting anti-apoptotic proteins namely Bcl-xL and Bcl2. Moreover, it can simultaneously promote the activation of pro-apoptotic markers [62]. Bax activation evokes apoptotic cell death by inducing mitochondrial pore formation, which then facilitates a release of cytochrome-C and cleavage of poly (ADP-ribose) polymerase. NERO treatment in DOX-injected rats downregulated Bax, active caspase-3, caspase-9, APAF-1, cleaved PARP, and cytochrome-C. Moreover, it upregulated Bcl2 and Bcl-xL. The downregulation of proapoptotic proteins and upregulation of antiapoptotic proteins demonstrate inhibited DOX-induced mitochondrial dysfunction, DNA damage, ATM activation, and apoptosis. This could be ascribed to its potent antioxidant property, which curbs the initiation and onset of oxidative stress in the myocardium.

Taken together, our study reveals that NERO has the potential to protect myocardium from DOX-induced acute cardiotoxicity by countering oxidative stress through enhanced Nrf2 signaling, the inhibition of inflammatory mediators and cytokines, as well as the amelioration of NF-κB/MAPK activation. Moreover, the study findings also demonstrate favorable modulation of apoptotic cell death pathways which supports the salvage of cardiomyocytes.

## 5. Conclusions

Our study demonstrated that NERO is a potent antioxidant that has anti-inflammatory and antiapoptotic effects in acute cardiotoxicity, especially when induced by DOX. Furthermore, we identified numerous cell signaling mechanisms involved in attributing antioxidant, anti-inflammatory, and antiapoptotic effects, all of which mediate the upregulation of Nrf2/Keap1, suppression of NF-κB/MAPK, and favorable modulation of cell death pathways. Thus, it can be concluded that NERO or plants containing NERO could be important for improving the therapeutic usefulness of DOX in chemotherapy by exerting cardioprotective effects.

## Data Availability

The data presented in the present study will be available from the corresponding author upon reasonable request.
